# Advances in Diagnostic Bronchoscopy

**DOI:** 10.3390/diagnostics11111984

**Published:** 2021-10-26

**Authors:** Yi-Cheng Shen, Chia-Hung Chen, Chih-Yen Tu

**Affiliations:** 1 Division of Pulmonary and Critical Care Medicine, Department of Internal Medicine, China Medical University Hospital, Taichung 40447, Taiwan; greywolf0127@gmail.com; 2 Graduate Institute of Clinical Medical Science, China Medical University, Taichung 40447, Taiwan; 3 School of Medicine, China Medical University, Taichung 40447, Taiwan

**Keywords:** bronchoscopy, endobronchial ultrasound, navigation bronchoscopy

## Abstract

The increase in incidental discovery of pulmonary nodules has led to more urgent requirement of tissue diagnosis. The peripheral pulmonary nodules are especially challenging for clinicians. There are various modalities for diagnosis and tissue sampling of pulmonary lesions, but most of these modalities have their own limitations. This has led to the development of many advanced technical modalities, which have empowered pulmonologists to reach the periphery of the lung safely and effectively. These techniques include thin/ultrathin bronchoscopes, radial probe endobronchial ultrasound (RP-EBUS), and navigation bronchoscopy—including virtual navigation bronchoscopy (VNB) and electromagnetic navigation bronchoscopy (ENB). Recently, newer technologies—including robotic-assisted bronchoscopy (RAB), cone-beam CT (CBCT), and augmented fluoroscopy (AF)—have been introduced to aid in the navigation to peripheral pulmonary nodules. Technological advances will also enable more precise tissue sampling of smaller peripheral lung nodules for local ablative and other therapies of peripheral lung cancers in the future. However, we still need to overcome the CT-to-body divergence, among other limitations. In this review, our aim is to summarize the recent advances in diagnostic bronchoscopy technology.

## 1. Introduction

The early detection of lung nodules for diagnosing curable lung cancer is very important [1,2]. The National Lung Screening Trial demonstrated the utility of low-dose computed tomography (CT) scans to increase early detection and reduce mortality from malignant tumors [3,4]. This trial reported 39.1% of subjects in the low-dose CT screening group as having at least one positive result [3]. Approximately 80% of these lesions were peripheral pulmonary lesions (PPLs), according to the National Lung Screening Trial and the NELSON trial. The increase in the incidental discovery of pulmonary nodules has led to more urgent requirement of tissue diagnosis [3,5].

There are various modalities for the diagnosis and tissue sampling of pulmonary lesions, but most of these modalities have their own limitations. CT-guided transthoracic needle aspiration has a higher yield for peripheral small lesions, compared with bronchoscopy, but faces difficulty in reaching lesions over central and mediastinum areas [6]. A previous study demonstrated that transthoracic biopsy for malignant lesions has 93% sensitivity, 100% specificity, and a 25% complication rate; pneumothorax accounted for 24% of complications [7]. Conventional bronchoscopy has lower sensitivity for malignant lesion diagnosis, and is poorer for lesions of less than 20 mm in diameter [7,8]. Although conventional bronchoscopy has a lower diagnostic yield than CT-guided transthoracic needle biopsy (TTNB) for the diagnosis of peripheral pulmonary lesions, the risks of complication are significantly lower.

This has led to the development of many advanced technical modalities, which have empowered pulmonologists to reach the periphery of the lung safely and effectively. These techniques include thin/ultrathin bronchoscopes, radial probe endobronchial ultrasound (RP-EBUS), fluoroscopy, and navigation bronchoscopy—including virtual navigation bronchoscopy (VNB) and electromagnetic navigation bronchoscopy (ENB). Recently, newer technologies—including robotic-assisted bronchoscopy (RAB), cone-beam CT (CBCT), and augmented fluoroscopy (AF)—have been introduced to aid in navigation to PPLs. Technological advances will also enable more precise tissue sampling of smaller peripheral lung nodules for local ablative and other therapies of peripheral lung cancers in the future.

PPLs are defined as lesions located in the bronchi that are not visible under bronchoscopy [9]. Diagnosis of PPLs suspected of malignancy remains a challenge. However, there are no clear guidelines for these various endobronchial modalities. The American College of Chest Physicians’ lung cancer guidelines recommend minimally invasive modalities, and take factors such as lesion characteristics, patient’s condition, physician’s skill, and complications into account [3,5,10,11,12,13,14,15]. Various navigational bronchoscopies have been developed for PPLs, increasing the diagnostic yield. Minimally invasive transbronchial treatment may also work by using more accurate three-dimensional (3D) imaging in the future [16,17].

Several factors may increase the diagnostic yield of transbronchial needle aspiration, such as lesion size of more than 3 cm, upper and middle lesion location, the presence of the CT bronchus sign [18,19,20,21,22,23,24], operator skill, adequate sampling tools, use of real-time sampling guidance, rapid on-site evaluation (ROSE), and the type of sedation used for the procedure [25,26,27,28,29,30].

Nodule size is an important factor in diagnostic yield [21,31,32]. However, Seijo et al. reported that lesion size (<3 cm vs. >3 cm and <2 cm vs. 2–3 cm) did not seem to show a statistically significant difference, which is also consistent with the findings of the NAVIGATE study and Chavez et al. [33,34,35]. The diagnostic yield of different locations (upper lobe vs. middle lobe vs. lower lobes) did not show a statistically significant difference; this result is consistent with the findings of the NAVIGATE trial [33,34]. The American College of Chest Physicians’ guidelines for the diagnosis of lung cancer also revealed that the sensitivity of bronchoscopy for diagnosing lesion in peripheral lung was lower than that in central lung [36].

Seijo et al. and the NAVIGATE study also reported the presence of the bronchus sign as a significant factor in diagnostic yield. [33,34] However, a retrospective study revealed no significant difference in diagnostic yield with and without the CT bronchus sign [30]. The experience of the operator is also a factor affecting the diagnostic yield in some studies [29,31], but this was not consistent in another cohort [30]. Moreover, a lower diagnostic yield (64.9%) was noted in centers with low procedural volumes (0–4 cases/month) in the NAVIGATE study [34], but another study showed that this factor does not affect the diagnostic yield [30].

A retrospective review based on the Tsuboi classification reported better yields and sensitivity with types I and II compared to type III; this result was consistent with the findings of Tsuboi et al. [37]. In Tsuboi type I (the bronchus ends in the lesion) and Tsuboi type II (nodule in the bronchus), the instruments can directly reach the lesion. In Tsuboi type III (lesion compressing the bronchus), the instruments cannot get through the compressed bronchus, making the diagnosis more difficult. It is possible that transbronchial sampling instruments will be better able to reach these lesions in the future [38]. The bronchoscopic diagnostic yield for PPLs is 53–65% using various sampling tools [39]. After the fifth biopsy specimen being sampled, the cumulative diagnostic yield reaches a plateau. Therefore, at least five biopsy specimens and washing after brushing are required in order to improve the diagnostic yield [21,40]. Furthermore, moderate sedation has also been found to affect diagnostic yield in a meta-analysis [27]; this cohort revealed that the use of conscious sedation in patients can result in better diagnostic yield, consistent with the study by Bowling et al. [28].

More recently, CT-to-body divergence has been put forward as a concept of the difference between pre-procedure planning CT and actual navigational bronchoscopy. The divergence of the mapping guided by navigational software, the true anatomy, the lung volumes, and the position of the target found during bronchoscopy may be caused by factors including atelectasis, nodule movement with respiration motion, and the physical changes between the planning phase and the procedure [16,41,42]. In this review, our aim is to summarize the recent advances in navigational bronchoscopy technology.

In 1966, Professor Shigeto Ikeda, the father of flexible bronchoscopy, introduced to the world the first flexible bronchoscope [43]. Professors Becker, Hurter, and Hanrath invented the RP-EBUS probe for target lesion confirmation prior to biopsy in 1992–1996 [44,45], and Professor Noriaki Kurimoto presented the guide sheath system of the RP-EBUS probe in 2004.

## 2. Endobronchial Ultrasound (EBUS) and a Guide Sheath (GS) (1992–1996, 2004) 

According to the American College of Chest Physicians’ guidelines, RP-EBUS-guided lung biopsy can be used as an important means of diagnosis of PPLs, and should be prioritized [46].

Kurimoto et al. introduced a technique of EBUS with a guide sheath (EBUS-GS) to improve the diagnostic yield of PPLs (Supplementary Material Figure S1). The overall diagnostic yield of the EBUS-GS was 77% [47].

Pulmonologists can check the relative position of PPLs and instruments, and perform sampling under visualization by using the RP-EBUS. When the wire arrives at the target, the physician removes it and inserts the probe of RP-EBUS in the GS, while keeping the GS in place. After localization, the radial probe is withdrawn and the sampling instruments are inserted into the GS, and specimens can be collected [47]. A systemic review and meta-analysis indicated good performance of RP-EBUS-guided lung biopsy compared with the conventional bronchoscopic lung biopsy modality [32].

The diagnostic yield was affected by the lesion size and probe location [32]. A meta-analysis revealed that the diagnostic yield was better for lesions with a diameter larger than 20 mm than for the lesions with a diameter of less than 20 mm. Many studies showed that positioning the probe within, adjacent to, and outside the lesions has the highest, lower, and lowest diagnostic accuracy, respectively [21,35,48,49,50]. The overall complication rate of RP-EBUS in the diagnosis of PPLs was 1.08–2.8%, including 0.8–1.4% pneumothorax, 1.1% bleeding, and 0.3–0.5% pneumonia [32,51,52,53,54].

## 3. Ultrathin Bronchoscopy (UTB) (1996) 

The conventional bronchoscope is limited by its large size, and cannot reach PPLs in more distal locations. The first thin bronchoscopy for PPLs in adult patients was invented in 1885 [55]. An ultrathin fiberscope with a 2.7 mm external diameter and 0.8 mm internal diameter, with a working channel, was introduced in 1996. The ultrathin bronchoscope can be used through the tracheal tube during mechanical ventilation [56]. However, the smaller working channel of the pediatric bronchoscopes limits the instrument to adequate and high-quality specimens. A prospective randomized control trial compared the UTB with a 2.8 mm outer diameter (OD) and 1.2 mm working channel with a standard-size bronchoscope, and the diagnostic yield of the UTB was lower than the yield of the standard-size bronchoscope. UTB was not better than standard bronchoscopy, possibly because of the small working channel, which is too small to obtain enough high-quality specimens [57]. Moreover, the ultrathin bronchoscope can reach more distal areas, but the location cannot be directly visualized through bronchoscopy, and needs to be confirmed by fluoroscopy or RP-EBUS. To break this limitation, ultrathin bronchoscopes with a 2.8–3.5 mm OD and proper-sized working channel have been developed to allow the use of RP-EBUS and more elasticity to pass smaller airways. The study revealed that the 3 mm OD ultrathin bronchoscope with a 1.7 mm working channel (Figure S2), compared with the 4 mm OD conventional bronchoscope, can reach more distal areas and has a better diagnostic yield [58]. A retrospective study also demonstrated that the diagnostic yield of RP-EBUS was significantly higher in ultrathin bronchoscopy without a guide sheath than in conventional bronchoscopy with a guide sheath, both under fluoroscopic guidance. There was no significant difference in the complication rate between the two groups [59].

## 4. Virtual Bronchoscopic Navigation (VBN, VNB) (2002) 

Virtual bronchoscopy (VB) is not an endoscopic procedure; it is an imaging modality that utilize non-contrast-enhanced computed tomographic (CT) images to reconstruct 3D airway images that appear similar to those visualized during invasive bronchoscopy. Virtual bronchoscopic navigation (VBN) uses virtual bronchoscopy CT imaging to guide the bronchoscope to PPLs in the lungs (Figure S3).

A meta-analysis revealed that navigation bronchoscopy has a significantly higher overall diagnostic yield than non-navigation bronchoscopy for PPLs [18]. Another systematic review and meta-analysis of randomized control trials revealed contrasting findings, which demonstrated that the overall diagnostic rate was similar in VBN-assisted (VBNA) and non-VBN-assisted (NVBNA) groups [60]; nevertheless, the VBNA group had significantly shorter total examination time compared with the NVBNA group; this result was consistent with the findings of Xu et al. and Liu et al. [49,61]. Furthermore, the sub-analysis also revealed the superiority of VBNA over NVBNA among patients with PPLs less than 20 mm in diameter, but the subgroup analysis for lesions more than 20 mm in size revealed the diagnostic yield to have no significant difference between the two groups. The study by Xu et al. including 105 patients showed no significant difference between the presence or absence VNB under the EBUS group, whether lesion size was less than 20 mm or 20–30 mm [49]. A study including 129 patients compared different VBN software, and the results revealed that VINCENT version 5.5 better reproduced peripheral bronchi than the LungPoint system [62].

Despite experience with numerous bronchoscopic techniques, numbers of peripheral pulmonary lesions still present significant challenges for interventional pulmonologists. Electromagnetic navigation bronchoscopy (ENB) systems were introduced to overcome such limitations. Schwarz et al. [63] first demonstrated their acceptable diagnostic yield (69%) and safety by using an ENB system (superDimension) for diagnosing PPLs.

## 5. Electromagnetic Navigation Bronchoscopy (ENB) (2005)

ENB utilizes a planning phase CT scan to reconstruct a virtual bronchoscopic image with an additional navigational tool—an electromagnetic field—which provides dynamic and temporally tracked guidance during bronchoscopy. ENB has been used in clinical practice since 2005, but more widely used in recent years [63,64]. The American College of Chest Physicians’ guidelines for the diagnosis of lung cancer specifically state the increased yields and safety of ENB, and recommend ENB as the preferred diagnostic modality for suspicious PPLs if equipment and expertise is available [36,65]. The US National Comprehensive Cancer Network (NCCN) guidelines also mention that navigational bronchoscopy may benefit patients with peripheral nodules. [66] Electromagnetic navigation bronchoscopy (ENB) systems rely on a magnetic field around the patient to detect a tracked device in order to obtain the position overlaid on the virtual bronchoscopic map. This is an integration of an electromagnetic tracking system and VBN, which includes a virtual pathway as a map during bronchoscopy, reconstructed by using pre-procedure high-resolution three-dimensional (3D) CT imaging. The system can synchronize the imaging to the EM field via selected points and provide a route to the lesion. Then, the physicians can track the synchronized probe during bronchoscopic navigation. The probe in the working channel can further pass the tip of the scope into smaller areas, and drive along the map to reach the target [67]. Then, the operator can use RP-EBUS and fluoroscopy for real-time confirmation of the target location before sampling instrument insertion. In addition to specimen sampling, fiducial markers placement and dye marking can also work via this system.

There are two commercially available ENB systems—the superDimension^TM^ System, and the SPiN Thoracic Navigation System^TM^—in the USA. Both systems need pre-procedure CT imaging and overlay the electromagnetic field on reconstructed four-dimensional (4D) CT scan imaging. These two systems have distinct advantages, depending on the different setting and lesion characteristics. When using the superDimension™ system, the procedure can be performed in the operating room or the endoscopic room. When using the SPiN Thoracic Navigation System™, because room mapping is not needed, the procedure can be carried out in either of these rooms.

## 6. SuperDimension^TM^ System 

The superDimension system utilizes a static inspiratory breath-hold CT chest scan for the planning phase. A sensor-locatable guide is tracked on the navigation system, synchronized with reconstructed CT imaging, and is inserted into an extended working channel (EWC) through a bronchoscope (Figure S4).

The EWC has various angles—including 45°, 90°, and 180°—to offer elasticity during navigation and remain in site after the locatable guide is shifted to the sampling instruments.

## 7. SPiN Thoracic Navigation System^TM^

The SPiNDrive system uses inspiratory- and expiratory-phase chest CT imaging during the planning phase to observe lesion movement due to the respiratory cycle. The electromagnetic generator is mobile, and can be placed in different locations. The system incorporates skin-tracking pads with electromagnetic sensors. These pads are put on the patient’s chest wall, and can track the patient’s breathing motion and help to reconstruct a 4D map for navigational bronchoscopy and sampling guidance [68,69]. The SPiNDrive system has combination of a locatable guide and EWC, and the use of a tip-tracked biopsy instrument allows continuous direct navigation.

Moreover, there is the SPiN Perc™ system, which can help the physician to conduct percutaneous or transthoracic needle aspiration (TTNA) biopsies in the same procedure. This modality is used for patients with lesions located too peripherally in the lung(s) to be reached. Several studies demonstrated diagnostic a yield increase from 70 to 80% by using this approach, with 17% and 21% pneumothorax rates, respectively; the rate of pneumothorax was higher than with bronchoscopy, and similar to CT-guided TTNA [68,69,70]. A multicenter prospective trial using ENB with TTNA has been conducted, and the results are pending [71]. Both the superDimension and SPiN systems can be used for more accurate localization and dye-marking of PPLs. The SPiN platform also can use the SPiN Perc for PPLs near the pleural surface, or that are difficult to reach within the same procedure. These two systems provide a stabilization-assisted system, which is fixed to the procedure table with mobile ergonomic arm. The bronchoscope can be fixed to this system and stabilized in the immovable position when the target is reached. This can decrease the device movement and allow the scope and instrument to remain stable and more precise—especially during tissue sampling and instrument exchange [72].

## 8. LungCare Navigation System

The latest ENB system, LungCare, has been approved for clinical usage since 2016 in China. This system has a workstation with computer software, a locatable wire, a position detection wire, and an electromagnetic board, and can be used in combination with guide sheaths, puncture needles, and different sized bronchoscopes, because of its different types of locatable wires [73,74]. The position detection wire can sense respiratory movement, and provides continuous synchronization with the workstation during the procedure. Three position detection wires with three electrodes attached to a patient’s chest can transfer a signal to the workstation.

Technical problems with the stability and extension of the probe–instrument complex can exist—for example, catheter slippage under significant torque, or during instrument exchanges. Moreover, visualization at the distal divergence is not real-time, and makes bronchoscopy sharply angulated and subject to torque. However, ENB is still the most used modality to reach the PPLs, and as an alternative to transthoracic biopsy in patients with higher risk. Additionally, the location of peripheral nodules with indocyanine green injection for precise resection can also work with ENB [75,76].

Several meta-analyses reported 65–82.5% sensitivity for diagnosing PPLs, and 0–5% complications [27,34,77,78,79,80,81]. While previous studies have reported efficacy and safety, with a low complication rate, in the diagnosis of PPLs using ENB, the technique highly depends on operator skills and anatomy. Several studies have reported larger lesion size, upper and middle lobe lesions, presence of the bronchus sign, and lesion visualization via RP-EBUS, and concurrent use of ENB with RP-EBUS has been reported to improve diagnostic yield [27,79,80]. The registered data of the AQuIRE Registry revealed that several centers have worse diagnostic yield with ENB (38.5%) compared to RP-EBUS (57%), which may be because specialized centers have better efficacy than the community [39]. A single-center retrospective study revealed a lower diagnosis rate, which may have been caused by the type of sedation [31]. Respiratory variation, coughing, respiratory distress, and other irritations during the procedure may affect the diagnostic yield [82]. Most previous ENB studies reported higher diagnostic yield for patients undergoing the procedure with general anesthesia [29,77,79,83,84], which suggests that the sedation quality during ENB can affect diagnostic yield. However, Cherian et al. recently reported that ENB has high diagnostic yield and safety even when performed by an operator without formal training and in low-resource settings under moderate sedation [30].

The prospective multicenter NAVIGATE study revealed a 73% diagnostic yield with a pneumothorax rate of 4.3% and hemorrhage rate of 2.5%. Multivariate predictors of better diagnostic yield include the use of less than three sampling tools, lymph nodes biopsied, the bronchus sign being present, multiple lesions being biopsied, and a procedure time of less than 60 min. [34] Another systemic review and meta-analysis of 40 studies and 3342 participants reported a pool sensitivity of 77% and a specificity of 100% for malignancy, with a receiver operative characteristic of 0.955 and pneumothorax rate of 2.0%. The mean distance from the sensor tip to the center of the lesion, the number of samples, and the cancer prevalence affect sensitivity [81].

It is worth mentioning that a cohort study revealed that ENB is more cost-effective when the likelihood of an accurate diagnosis is equal to that of TTNA. This can happen in certain subgroups in whom TTNA is unlikely to achieve an accurate diagnosis, or is performed by experienced operators to reach a high accuracy with ENB [85].

ENB systems elevate the potential for PPL diagnosis and sampling; however, they lack peripheral visualization during navigation. This led to the introduction of robotic bronchoscopy. The robotic system uses a virtual map generated from reconstructed 3D CT and EM field mapping as an ENB system, along with advanced robotic arms.

## 9. Robotic Bronchoscopy

Robotic bronchoscopy systems are the newest technique and least invasive modality for the diagnosis of PPLs. There are two systems, including the Monarch™ Platform (Auris Health©, Redwood City, CA, USA, FDA approved in March 2018) and the Intuitive Ion™ robotic platform (Intuitive Surgical©, Sunnyvale, CA, USA, FDA approved in February 2019) [68,86,87]. These systems can reach more peripheral areas of the lungs, have more delicate control and navigation to the target, and show more stabilization during tissue sampling—especially when the target is an eccentric lesion via peripheral TBNA. In addition, because the robot-assisted system uses a smaller bronchoscope, it also has direct visualization for more distal areas [68,88]. The two systems both have similar equipment, including the bronchoscope, robotic arms, a tower, and a controller.

## 10. The Monarch™ Platform 

The Monarch^TM^ system (Auris Health©, Redwood City, CA, USA) is composed of a 6.0 mm, 130° articulating outer sheath and a 4.4 mm bronchoscope with a 2.1 mm working channel that can flex 180° in any direction beyond the sheath. With the use of a handheld controller, it allows the pulmonologist to navigate the tortuous and more distant areas of the lungs, along with continuous visualization. All parts of the scopes can independently lock into position for stability during instrument exchange and tissue sampling. The operator can control the instrument more precisely and guide it further, to the periphery of the lung lesions, compared to the conventional bronchoscope [89]. The Monarch^TM^ system uses similar equipment to ENB, including direct visualization, real-time and virtual bronchoscopy, electromagnetic navigation, and robotic real-time data that can precisely localize the bronchoscope. Fluoroscopy and RP-EBUS can also be integrated within the system to confirm more precise positioning during procedures [88,89,90].

A retrospective multicenter study using the Monarch™ system in 165 patients with 167 lesions demonstrated successful navigation of 88.6% and diagnostic yield of 69.1–77% for the lung nodules. The average size of target lesions was 25.0 ± 15.0 mm. A total of 71% of the lung nodules were located in the outer third of the lung, and 63.5% of patients had a bronchus sign. The yields of concentric, eccentric, and absent RP-EBUS views were 81.5, 71.7, and 26.9%, respectively. The rate of pneumothorax was 3.6%, 2.4% required chest tube placement, and airway bleeding was 2.4% [88,91]. A prospective multicenter pilot and feasibility study (BENEFIT) using the Monarch™ system reported success rate of 96.2% for lesion localization using RP-EBUS, and a diagnostic yield of 74.1% in 54 patients. Median lesion size was 23.0 mm (IQR, 15 to29 mm), and 59.3% of patients had a bronchus sign. Peripheral lesions with a concentric RP-EBUS view had a diagnostic yield of 80.6%, and those with an eccentric view had a yield of 70%. The diagnostic yield of eccentric lesions was significantly higher than the previously reported yield of 30–40%. [21] The rate of pneumothorax was 3.7%, and the rate of requiring tube thoracostomy was 1.9%. [92] Another prospective multicenter study (TARGET) using the Monarch™ Endoscopy Platform is currently enrolling patients, and aims to enroll 1200 patients at up to 30 investigative sites to evaluate the clinical safety and diagnostic accuracy of robotic-assisted bronchoscopy with biopsy [88,93].

## 11. Intuitive Ion™ Robotic Platform 

The Ion^TM^ endoluminal system (Intuitive Surgical©, Sunnyvale, CA, USA) uses fiber-optic shape-sensing in conjunction with real-time and virtual bronchoscopy. The scope consists of an ultrathin, fully articulating catheter with a 3.5 mm outer diameter, 2.0 mm working channel, and a vision probe in the working channel. The catheter includes fiber-optic shape sensors that provide real-time, accurate location and shape feedback. The system can keep the catheter in its current formation and reach further lesions stably. The optical probe is withdrawn upon reaching the target, and the instrument can be inserted in the remaining sheath. This system provides a custom-designed flexible needle (Flexision™, Intuitive Surgical©, Sunnyvale, CA, USA) that can be passed through the positioned catheter, which can be advanced through tortuous airways with smaller radii, and can then be deployed into the target lesion in a direct fashion [68,88]. The user operates the system via a trackball and scroll wheel, which are combined with a distal tip articulation system to provide direct visualization during the navigation process. Navigational bronchoscopy, RP-EBUS, and fluoroscopy can be integrated into the tower systems [94].

Fielding et al. performed the first human feasibility study using the Ion^TM^ endoluminal system on 29 patients with a mean lesion diameter of 12.2 mm in the axial plane, and reported 96.6% localization and tissue sampling success. The overall diagnostic yield and diagnostic yield for malignancy were 79.3% and 88%, respectively. The CT bronchus sign was present in 58.6% of cases, and approximately half of the cases had an eccentric RP-EBUS view. The high yield—especially for the eccentric RP-EBUS view—was attributed to the ability to visualize peripheral airways and to deploy the TBNA needle perpendicular to the airway towards the lesion. No pneumothorax or major bleeding were encountered. However, procedure times averaged 95 min initially, and then shortened to 61 min [87]. A prospective single-arm multicenter clinical study (PRECIsE) using the Ion^TM^ endoluminal system is in progress, and is expected to enroll 360 patients, with the primary outcomes assessing navigation and biopsy success, and secondary outcomes assessing complications [95].

Due to the robotic systems retooling the bronchoscope into one with accurate motion, adjustable angulation, and better stability, the robotic platforms have the ability to potentially overcome some limitations of the currently available guided bronchoscopy systems, and increase the diagnostic yield for PPLs—especially for eccentric RP-EBUS views [87,91,92]. These new RAB platforms with sheaths and scopes with fiber-optic shape sensing can reach further areas up to the ninth generation of airways—compared to conventional bronchoscopy, which can only reach the sixth generation of airways—and have entire real-time visualization and mapping for tissue sampling and marking lesions for diagnosis and surgery [81]. The small outer diameter of the bronchoscope also helps the scope to be wedged and locked in the target segment, which can allow for tamponade and containment of the bleeding. Because of their precision in locating PPLs, the RAB platforms may guide bronchoscopic ablative therapies for treating oligometastatic lesions or inoperable peripheral lung tumors [90,96].

However, the most important limitations to overcome are the lung movement during the procedure and the CT-to-body divergence [97]. The navigational bronchoscopic techniques utilize pre-procedure planned CT imaging for navigational mapping, and cannot reflect the real state of the lung in real time during the procedure—especially while sampling tissue. The combination of the real-time imaging modality including cone-beam CT (CBCT) and augmented fluoroscopy (AF) can obtain real-time 3D imaging during the procedure to identify the lesion, scope, and instrument location. Furthermore, the weakness of this technique includes higher cost, more complex configuration in the procedure room, longer procedure time, and the need for a learning curve. We still need further research in order to evaluate the efficacy of robotic-assisted bronchoscopy. With more persistence, practice, and patience, robotic-assisted bronchoscopy may become the next stepping stone for the diagnosis and treatment of PPL.

A variety of bronchoscopic technologies for the diagnosis of PPLs have been developed in the past two decades. The diagnostic yield seems to have plateaued around 70%, and is often lower [27,34,39,84,91,98,99,100,101]. These modalities have failed to reach a consistently high diagnostic yield. Various factors affect them, including inaccurate real-time visualization and confirmatory technique, CT-to-body divergence, and poor-quality sampling. RP-EBUS is commonly used to confirm the lesion location, and can also be misled by atelectasis or hemorrhage [41]. CBCT can provide high-resolution, real-time, intraprocedural 3D imaging, and support the navigation, confirmation, and tissue sampling phases to correct for CT-to-body divergence, representing a feasible way to overcome those limitations mentioned above [16,41,102,103,104,105,106].

## 12. Cone-Beam Computed Tomography (CBCT) 

CBCT uses intraprocedural 3D imaging using a C-arm, and was introduced in the early 2000s; its use was quickly and widely adopted for various clinical applications, including pulmonary bronchoscopic intervention [107,108,109,110]. CBCT uses a compact CT system with a moving C-arm requiring volumetric data during the procedure to provide real-time information about the instrument and target lesion location (Figure S5).

CBCT is performed after anesthesia, and typically before the bronchoscope is inserted. The imaging reconstructed by projection imaging can be reformatted into coronal, sagittal, and axial views. Next, the target PPLs are located and outlined on those images by using dedicated softwar. [102,103,106,111]. Then, the lesion can be overlaid on live fluoroscopic imaging to provide a target for navigation and sampling.

The common trade names for CBCT include DynaCT (Siemens Healthineers, Germany), Innova CT (GE Healthcare, Waukesha, WI, USA), and XperCT (Philips Healthcare, The Netherlands). Fixed CBCT systems have types including floor, biplane, ceiling, and robotic; all of these can be used during bronchoscopy. The floor and biplane systems are less convenient because the base is fixed to the floor at the head end of the patient table, where the pulmonologist stands. Moreover, the system’s reach to the base of the lungs—especially in taller patients—could be limited depending on the C-arm depth, because the rotation point of the propeller is above the patient’s head. On the other hand, ceiling and robotic systems have friendlier configuration, better patient access, and less equipment re-arrangement during the switch between fluoroscopy and CBCT. Some mobile C-arm systems—including Cios Spin (Siemens Healthineers) and Vision RFD 3D (Ziehm Imaging, Florida, USA)—are also able to perform CBCT. Although mobile C-arms have a smaller field of view and a longer image acquisition time of ~30–60 s, they can move easily, and have a smaller footprint and more acceptable price [112].

Many studies have reported using CBCT to visualize PPLs during bronchoscopic procedures, including diagnosis, dye marking, and ablation [41,102,103,104,105,106,111,113]. During navigation bronchoscopy, CBCT can assist in navigation guidance and tool-in-lesion confirmation prior to sampling or ablation [41,102,103,104,105,106,111,113]. Furthermore, CBCT can detect atelectasis during procedures that may interfere with navigation, obscure the target location, and mislead the physician. In addition to a high positive end expiratory pressure, the use of CBCT can correct CT–body divergence caused by atelectasis. CBCT also can decrease CT–body divergence caused by respiratory movement or other factors by providing real-time navigational information.

CBCT also can be combined with other bronchoscopic techniques, such as ultrathin scopes, RP-EBUS, transbronchial instruments, and EMN [41,102,103,104,105,106,111,113]. The utility of CBCT is not to replace other navigation bronchoscopic techniques, but to provide additional confirmation and accurate real-time navigation, especially in the biopsy phase. Moreover, bronchoscopic ablation requires more precise locations of the instrument, the target, and vital structures, in which CBCT can play an important role [114].

Several studies have demonstrated that CBCT can be used to confirm the relative distance between the instrument and the target prior to biopsy, and has the potential to improve diagnostic yield with fewer needle repositions and reduced complications [41,103,104,105,115]. A prospective study by Hohenforst-Schmidt el al. showed that traditional bronchoscopy combined with CBCT has a navigational yield of 91% and a diagnostic yield of 70%. Moreover, CBCT-guided transbronchial biopsies (TBBs) for incidental solitary pulmonary nodules ≤ 2 cm found a sensitivity to malignancy of 82%, which is much better than fluoroscopy-guided conventional TBBs, with a mean value of 50% [102,116]. A small prospective pilot study of 20 patients demonstrated that CBCT-guided bronchoscopy resulted in 20% and 25% increases in the navigational and diagnostic yield, respectively, of thin/ultrathin bronchoscopy for PPLs, with an acceptable radiation dose ranging between 8.6 and 23 mSv [41]. A prospective single-center study of 87 patients with 107 lesions also reported that the addition of CBCT imaging to the electromagnetic navigation system increased navigation success from 52.2% to 87.5%, and diagnostic accuracy from 50% to 75% [117]. A multivariate analysis by Park et al. reported that tool-in-lesion confirmation by CBCT prior to biopsy was the only factor related to higher diagnostic yield [105].

In summary, CBCT plays an important role during bronchoscopic procedures by localization of the instrument and the target lesion, tissue sampling under real-time guidance, and decreasing CT-to-body divergence, including atelectasis caused by false positive RP-EBUS images. Several studies are ongoing to explore whether this technique can improve outcomes of bronchoscopy for PPLs. There are some limitations and drawbacks of using CBCT as an adjuvant modality in bronchoscopic procedures, including the radiation exposure, the complex workflow in most procedure rooms, and the longer procedure time due to the number of scans and preparation.

Precise localization and sampling are especially important for bronchoscopy. Previous traditional modalities including virtual navigation bronchoscopy, ENB, and robotic-assisted systems with shape-sensing can be combined with CBCT to acquire 3D images and localize targets, probes, and instruments, demonstrating a 91% localization rate but a 70% diagnostic yield [102]. This diagnostic yield has not increased in parallel with localization success.

These approaches all use a virtual navigational pathway guided by preoperative reconstructed CT images to guide the physician to the target. However, the navigation does not reflect the actual target location. A study revealed an average displacement distance of 13–22 mm compared to the expected location on the planning CT imaging. The distance can be larger than this, especially when evaluating lesions less than 20 mm in size. This can lead to missampling and lower diagnostic yield [118].

All of these bronchoscopic approaches are based on CT images in the planning phase, provide only a static overlay, and do not factor in CT-to-body divergence or respiratory movement.

Atelectasis also affects the visualization of the target. Pritchett et al. reported that atelectasis was identified to varying degrees in approximately 50% of their procedures [119]. Casal et al. [41] reported that the target lesion was totally obscured by atelectasis in 20% of their subjects. Atelectasis can be caused by various factors, including anesthesia, mucus plugs, chest muscle weakness, and hyperoxia [120], and can cause significant displacement of the target. The use of general anesthesia with a tidal volume of 8–10 mL/kg of ideal body weight and a positive pressure ventilation of at least 10 cm H_2_O can minimize atelectasis and reduce CT-to-body divergence [121].

Respiratory movement makes lung volume, airway orientation, and instruments’ location within the lungs change constantly. The overlay of the true lesion location and the target on the navigation guide only occurs at a single point in the respiratory cycle. This mismatch distance between full inspiration and end exhalation on pre-procedure chest CT scans has been reported to average 17.6 mm over the whole lung, and the average of the range near the base of the lung can reach up to 25.3 mm. This mismatch can significantly affect the diagnostic yield of ENB [42], which produces a high localization rate, but without consistent diagnostic yield [39,79,97,122,123]. The critical step of diagnostic bronchoscopy for PPLs is to localize the tip of the instrument within or at the border of the target; if we cannot overcome CT-to-body divergence and breathing motion, this can lead missampling of non-lesion sites.

At present, we need to aim to correct CT-to-body divergence using actual real-time visualization of the target, and improve the means of tracking lung motion during the bronchoscopic procedure for peripheral pulmonary lesions.

## 13. Augmented Fluoroscopy (AF) 

Fluoroscopic navigation is an adjuvant technique for the navigational bronchoscopy system. This modality utilizes tomosynthesis with continuous imaging to obtain multiple projections using a C-arm fluoroscopy machine in order to locate the target lesion (Figure S6). This makes real-time localization of the navigation probe more precise, and decreases the effect of CT-to-body divergence.

A prospective study by Hohenforst-Schmidt at al. first reported the use of airway structures overlaid on live fluoroscopy imaging during CBCT-guided bronchoscopy [124]. This technique—also termed “augmented fluoroscopy” (AF)—accurately provides real-time 3D imaging for every movement using the C-arm during the procedure. However, the overlaid images on the live fluoroscopy screen are static, and the true target location on the augmented fluoroscopic view can be obtained accurately only during breath holding.

By contrast, augmented fluoroscopy using the LungVision system can provide precise real-time target location during navigation and tissue sampling, with persistent dynamic tracing to the target.

## 14. LungVison^TM^ System

The LungVision™ system (Body Vision Medical Ltd., Ramat Ha Sharon, Israel, FDA approved in May 2017) is an image navigation system that integrates multimodal images and modalities of three-dimensional reconstructive maps generated from preoperative CT images, bronchoscopy, RP-EBUS, biopsy instruments, and intraprocedural real-time fluoroscopic visualization of airways to enable real-time augmented endobronchial fluoroscopic navigation, localization, and tissue sampling of PPLs. Guided pathways to the lesion are projected on the fluoroscopic screen in real time, directing the bronchoscopist to the highlighted target. Furthermore, the system has probe tracking along with fusional images, without the need for electromagnetic sensors. The software can adjust for motion during breathing and CT-to-body divergence via artificial intelligence [125]. Respiratory motion compensation enables the use of moderate sedation, with no need for general anesthesia or paralytic agents. The LungVision™ 2.0 system (FDA approved in May 2019) uses an image registration technique through powerful artificial intelligence algorithms, real-time tool-in-lesion confirmation through C-Arm-based tomography (CABT) technology—which is an alternative to CBCT—and navigation tool integration (RP-EBUS, EMN, etc.). Additional advantages are reduced cost and decreased radiation exposure, where the average radiation dose is 2.76 mSv, similar to low-dose CT. Previous reports have shown experience with this system [126,127,128].

A prospective analysis by Pritchett et al. reported that the average distance between lesion location measured by LungVision augmented fluoroscopy and actual location measured by CBCT was 5.9 mm. Lesion localization success rate and diagnostic yield were 96.1% and 78.4%, respectively. Diagnostic accuracy at 12 months follow-up was 88.2%. Diagnostic yield for lesions ≤ 2 cm in diameter was 70.6% [119]. Another prospective single-center study showed that the overall diagnostic yield, and that for lesions smaller than 20 mm, were 81.8% and 72.2%, respectively, with the LungVision system [129]. The diagnostic yield was better than those of the VBN and ENB navigation systems [18,27,80]. A multicenter study revealed 93% nodule localization success rate and 75.4% overall diagnostic yield by adding rapid on-site evaluation [130].

Augmented endobronchial fluoroscopic navigation with the LungVision system provides safe, feasible, and real-time lesion localization accuracy. Intraprocedural augmented fluoroscopy with real-time 3D CABT is an effective tool to assist in lung nodule biopsies. LungVision enables real-time nodule and pathway overlay on native fluoroscopy and, furthermore, allows real-time 3D CABT, similar to CBCT imaging, but requiring only a standard fluoroscopy C-arm.

## 15. Combination Study

Various technologies have been developed in the past two decades, each with its own advantages. The combination of multiple approaches can make the most of their respective advantages and achieve a higher diagnostic yield.

### 15.1. EBUS+VNB

A study revealed that the diagnosis yield of VBN in conjunction with EBUS was significantly higher than that of EBUS alone for lesions less than 2.0 cm in diameter. The ultrasound probe of EBUS could further confirm the target location in the VBN and EBUS group, and increased diagnostic yield to 70% [49]. Another study revealed that the combination of EBUS-GS and VNB systems (LungPoint) was useful for diagnosing small PPLs [48].

### 15.2. EBUS+ENB

A prospective randomized controlled trial reported that the combined use of RP-EBUS along with ENB has better diagnostic yield of 88% compared to either technology alone [131]. A single-center retrospective study also demonstrated that ENB combined with RP-EBUS biopsy for the diagnosis of PPLs was safe and effective [132].

### 15.3. EBUS+Robotic+Fluoro

A small, retrospective, single-center study of 10 patients reported 90% diagnostic yield using RP-EBUS, robotic-assisted navigation with the Ion platform, and multiplanar 3D fluoroscopy with a Cios-Spin mobile 3D C-arm. Navigation to the target was successful in all cases [133].

### 15.4. ENB+CBCT+AF

A single-center retrospective study including the combination of ENB, CBCT, and AF in 75 patients achieved a high diagnostic yield of 83.7%, even for very small lesions (median diameter 16.0 mm), with a diagnostic accuracy of 93.5% and low complication rates, with an acceptable amount of radiation dose [106].

### 15.5. ULTRATHIN+EMN+RP-EBUS+CBCT+AF

Various studies have demonstrated that the combination of multiple modalities—including CT and fluoroscopic capabilities [105], ultrathin bronchoscopy and RP-EBUS [41], EMN [106,134], and transthoracic needle aspiration [102]—can increase the diagnostic yield from 70% to 84% [41,102,105,106,134].

### 15.6. EMN+RP-EBUS+CBCT+AF

A prospective single-center study revealed that the use of CBCT and AF imaging in addition to the original configuration of EMN and RP-EBUS can significantly increase navigation success, and almost 90% of small lesions could be reached. However, the overall diagnostic accuracy of 72.4% did not increase with the consistently higher navigation success. A more effective tissue acquisition methodology may improve the overall diagnostic yield [117].

## 16. Conclusions

Prior studies have demonstrated that when technologies are used in combination, the diagnostic yield of PPLs incrementally increases. That is to say, such technologies are not replacements but, rather, complementary to one another in combination. This improved diagnostic value and precise localization are vital for the development of bronchoscopic therapeutic techniques for PPLs, including local ablation in the future.

Numerous studies have used the superDimension™ platform. However, emerging evidence has demonstrated better results with the use of the SPiN Thoracic Navigation System™ and SPiN Perc™, Monarch™, and the Intuitive Ion™. Robotic-assisted systems can maintain stability during instrument exchange and tissue sampling. In Table 1, we briefly review the diagnostic yields and complication rates of studies using these systems (Table 1).

RP-EBUS-GS can further confirm the relative position (within, adjacent, or outside) of the target lesion and instrument when the navigation shows that the target has been reached, and exchange instruments along the GS to maintain the right pathway.

CBCT plays an important role in bronchoscopic procedures by identifying the relative locations of the instrument and the target lesion during sampling under real-time guidance, and decreasing CT-to-body divergence.

Augmented fluoroscopy using the LungVision system can localize target lesions and sample tissue more accurately under persistent real-time navigation and dynamic tracing to the target.

Sampling tools are selected according to the characteristics and location of the PPL. The sampling needs to obtain enough specimens for genetic analysis. The 1.5 mm forceps are relatively small and are widely used. Standard biopsy forceps or ultrathin cryoprobes are recommended for ground–glass opacity lesions due to the need for larger volume specimens. The puncture needle is recommended while the instrument is not within or adjacent to the PPL.

The ROSE can increase the sensitivity, improve the diagnostic yield, and shorten the operation time for RP-EBUS in the diagnosis of PPLs, and provides feedback on the specimens obtained during the procedure [138,139].

Cost, lesion accessibility, and pulmonologist experience should be considered when selecting these modalities for identifying the least invasive approach.

Moreover, bronchoscopic ablation should be performed only if we can accurately and reproducibly localize the target, obtain enough specimen for diagnosis, and stay at the center of the lesion for long enough to complete the ablation therapy. This necessitates a complete change in the way we use this technique or those of other fields, and will require further innovation and collaboration.

## Figures and Tables

**Table 1:** Clinical trial data.

	First Author [Ref.]	Year	N	Study Design	Diagnostic Yield %	Complication Rate%	Additional Technique(s)
Ultrathin							
	Oki et al. [58]	2015	310	Randomized trial	74%	3%	EBUS, Fluoroscopy, VBN
	Sumi et al. [59]	2020	168	Retrospective study	74.5%	3.9%	EBUS
VBN							
	Giri et al. [60]	2021	1626	Systematic review and meta-analysis	74.17%	-	EBUS-TBLB
	TAMIYA et al. [48]	2013	68	Retrospective study	Thin bronchoscopy with EBUS-GS under LungPoint guidance for small (≤30 mm) PPLs was 77.9%	-	LungPoint system + EBUS-GS Fluoroscopy
	Xu et al. [49]	2020	105 (50 VBN + 55 EBUS)	RCT	76.0% VBN+EBUS 65.5% EBUS	8.0% (4/50) VBN+EBUS 21.8% (12/55) EBUS	EBUS
	Liu et al. [61]	2020	202	Retrospective study	84.2%	-	EBUS-GS
	Kitamura et al. [62]	2021	131	Retrospective study	76.8%	No serious complications	EBUS-GS
ENB							
	Yang et al. [76]	2021	47 (35 percutaneous injection 12 ENB)	Prospective study	Location success rate 94.3% (33/35) vs. 100% (12/12)	Percutaneous marking group; 14% (5/35) pneumothorax	Fluoroscopy
SuperDimension™							
	Tian et al. [135]	2020	157 (105 CT-guided hook wire 52 ENB)	Retrospective study	Location success rate 94.3% (99/105) vs. 100% (52/52)	CT-guided localization group; 7.6% (8/105) aymptomatic hemopneumothorax, 3.8% (4/105) symptomactic hemopneumothorax, 0.9% (1/105) hemotysis, 0.9% (1/105) decoupling	
	Schwarz et al. [63]	2006	13	Prospective, controlled clinical study	69.2%	No device-related adverse events	
	Folch et al. [34]	2019	1215	Prospective multicenter cohort study	73%	4.3% pneumothorax, 2.5% hemorrhage	91% Fluoroscopy 57% EBUS
	Patrucco et al. [77]	2018	113	Retrospective observational study	69%	No procedural complications	Fluoroscopy-guided, ROSE
	Sun et al. [78]	2017	40	Prospective study	82.5%	No complications	EBUS and Fluoroscopy
	Gex et al. [79]	2014	1033	Systematic review and meta-analysis	55.7–87.5% Pooled 64.9%	3.1% pneumothorax, 1.6% tube thoracostomy	EBUS, Fluoroscopy, ROSE
	Zhang et al. [80]	2015	1106	Meta-analysis	60.0–94.0%	No procedural complications	EBUS, Fluoroscopy, ROSE
	Folch et al. [81]	2020	3342	Systematic review and meta-analysis	Overall sensitivity 78%	2.0% pneumothorax	95% superDimension EBUS, Fluoroscopy, ROSE
	Ost et al. [39]	2016	581 15 centers	Registry	EMN 38.5% EBUS 57.0% No EMN and no EBUS 63.7% EMN+REBUS 47.1%	1.7% pneumothorax, 0.2% bleeding, 0.2% refractory hypoxemia, 0.2% respiratory failure	80.8% (252/312) superDimension
	Becker et al. [82]	2005	29	Prospectively	69%	3.4% pneumothorax, 10.3% minor, self-limiting bleeding	EBUS
	Lamprecht et al. [29]	2012	112	Single-center, prospective, observational study	Overall 83.9%; The first 30 procedures 80%; The last 30 procedures 87.5%; Lesions ≤ 20 mm 75.6% Lesions > 20 mm 89.6%	1.8% pneumothorax	PET/CT and ROSE
	Mohanasundaram et al. [83]	2013	47	Retrospective analysis	89.4%	13% (6/47) pneumothorax	ROSE
	Khandhar et al. [84]	2017	1129	Prospective, multicenter study	Navigation success 91.8% (1036/1129)	3.2% (32/1000) CTCAE Grade ≥ 2 pneumothorax, 4.9% (49/1000) any-grade pneumothorax, 1.0% (10/1000) CTCAE grade ≥ 2, and 2.3% (23/ 1000) overall bronchopulmonary hemorrhage, 0.6% (6/ 1000) CTCAE Grade ≥ 4 respiratory failure	54.3% (543/1000) EBUS 90.1% (1017/1129) fluoroscopy ROSE
	Cherian et al. [30]	2021	76	Retrospective chart review	80.2%	1.3% (1/76) pneumothorax requiring tube thoracostomy	Fluoroscopy
	Bhatt et al. [136]	2018	285 (150 ENB, 150 TTB)	Retrospective cohort study	ENB 66.0% TTB 86.0%	Pneumothorax (tube) ENB 4.0% (2.7%, 4/150 requiring chest tube), TTB 28.7% (1.3%, 2/150 requiring chest tube), bleeding (symptomatic), ENB 3.3% (2%, 3/150, symptomatic), TTB 16.7% (1.3%, 2/150 symptomatic)	ROSE
	Eberhardt et al. [131]	2007	118 (39 EBUS only, 39 ENB only, 40 combined)	Prospective randomized controlled trial	69% EBUS only, 59% ENB only, 88% a combined	6% overall pneumothorax, 5% 2/39EBUS only, 5% 2/39ENB only, 8% 3/40 a combined	EBUS
	Wang et al. [132]	2021	37 (23 in the solid nodule group and 14 in the subsolid pulmonary nodule group)		91.8% (34 /37) diagnostic accuracy, 91.3% (21/23) solid, 92.8% (13/14) subsolid, 75% (27/36) diagnostic yield, 90.9% (20/22) solid, 50% (7/14) subsolid	2.7% (1/36) complications	EBUS
SPiN Thoracic							
Navigation System™	Oh et al. [31]	2021	100	Single-center retrospective study	53% upward trend after 60 cases, from 45–65%	16% (16/100) complications, 3% (3/100) pneumothorax, 4% (4/100) moderate bleeding	No use of additional equipment
	Belanger et al. [137]	2019	102 (56 ENB biopsy+FM placement, 37 biopsy, 9 FM placement)	Retrospective review	78%	2.9% (3/102) pneumothorax, 0.98% (1/102) hemorrhage, no complications occurred with FM placement using the EMN platform.	93 p’t under ENB +/− EMTTNA 65 p’t FM EBUS-TBNA ROSE
SPiN Perc™	MALLOW et al. [69]	2019	129	Retrospective, multicenter study	73.7%	17.8%	
	Yarmus et al. [70]	2016	24	Prospective single arm pilot study	The diagnostic yield for ETTNA alone was 83%, and increased to 87% (*p* = 0.0016) when ETTNA was combined with ENB. When ETTNA and NB were used with EBUS for complete staging, the diagnostic yield further increased to 92%	No bleeding events occurred. There were five pneumothorax (21%), of which only two (8%) subjects required drainage.	EBUS + ENB + ETTNA
Robotic							
Monarch™	Chaddha et al. [91]	2019	167	Retrospective multicenter study	69.1–77% navigation successful 88.6%	3.6% (6/167) pneumothorax, 2.4% (4/167) requiring chest tube, 2.4% (4/167) bleeding	EMN EBUS
	Chen et al. [92]	2021	54	Prospective multicenter pilot and feasibility study	74.1% localization success rate 96.2%	3.7% (2/54) pneumothorax, 1.9% (1/54) requiring chest tube	EBUS ROSE
Intuitive Ion™	Fielding et al. [87]	2019	29	Single-center	79.3% localization success rate 96.6%	No device-related adverse events	EBUS
AF							
LungVision™	Pritchett et al. [119]	2021	54	Prospective, single-center, single-arm	78.4% localization success rate 96.1%	No pneumothorax, respiratory failure, or bleeding events	ROSE Confirmed by CBCT
	Pertzov et al. [129]	2021	63	Prospective single-center	81.8%	1.6% (1/63) pneumothorax, requiring chest tube	50 p’t Cryobiopsy EBUS ROSE
	Cicenia et al. [130]	2021	57	Prospective multicenter study	75.4% localization success rate 93%		EBUS ROSE

## Supplementary Materials

The following are available online at https://www.mdpi.com/article/10.3390/diagnostics11111984/s1, Figure S1. The equipment and image of Endobronchial ultrasound, Figure S2. Flexible bronchoscopes, Figure S3. Virtual navigation images on LungPoint system, Figure S4. The Electromagnetic navigation image of superDimensionTM System, Figure S5. The Cone-beam computed tomography, Figure S6. The image of augmented fluoroscopy.
