# Supplementary material for: Advances in Diagnostic Bronchoscopy
Source: Diagnostics (Basel). 2021 Oct 26;11(11):1984. doi: 10.3390/diagnostics11111984 (PMC8620115; doi:10.3390/diagnostics11111984)

Figure S1. The equipment and image of Endobronchial ultrasound

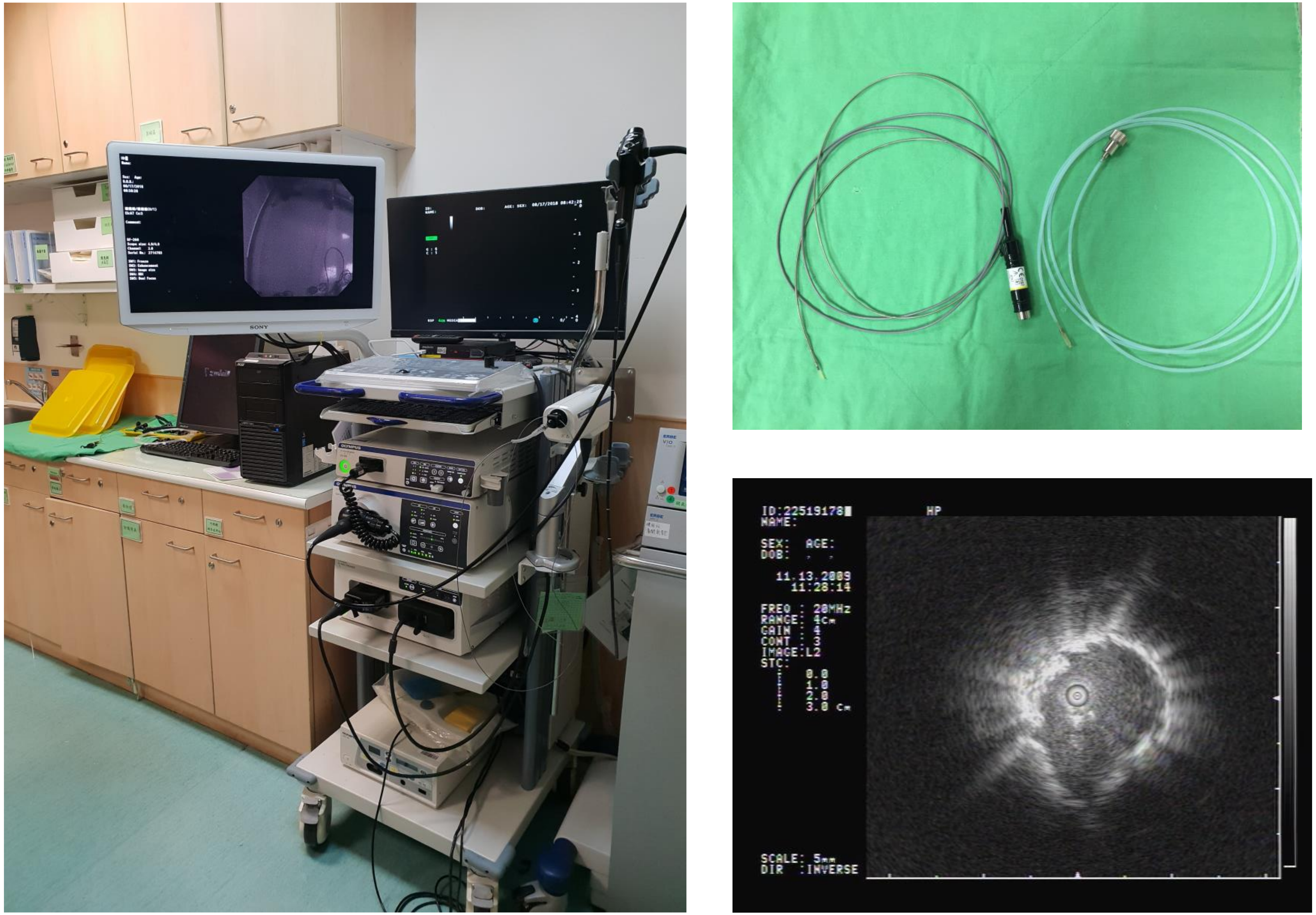

Figure S2. Flexible bronchoscopes. (A) a 3.0-mm ultrathin bronchoscope with a 1.7-mm channel; (B) a 4.8-mm bronchoscope with a 2.0-mm channel; and (C) a 5.9-mm bronchoscope with a 3.0-mm channel.

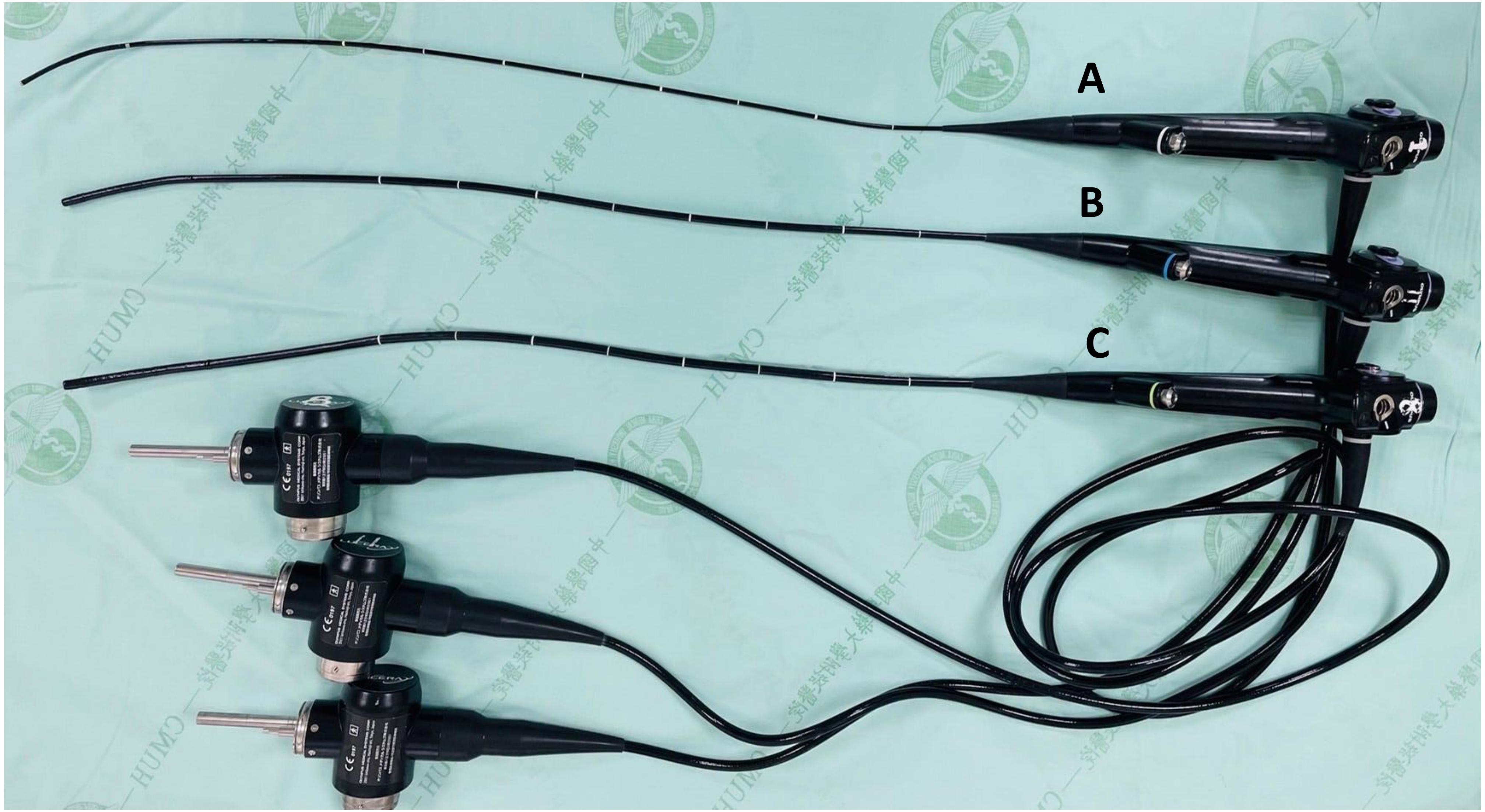

Figure S3. Virtual navigation images on LungPoint system

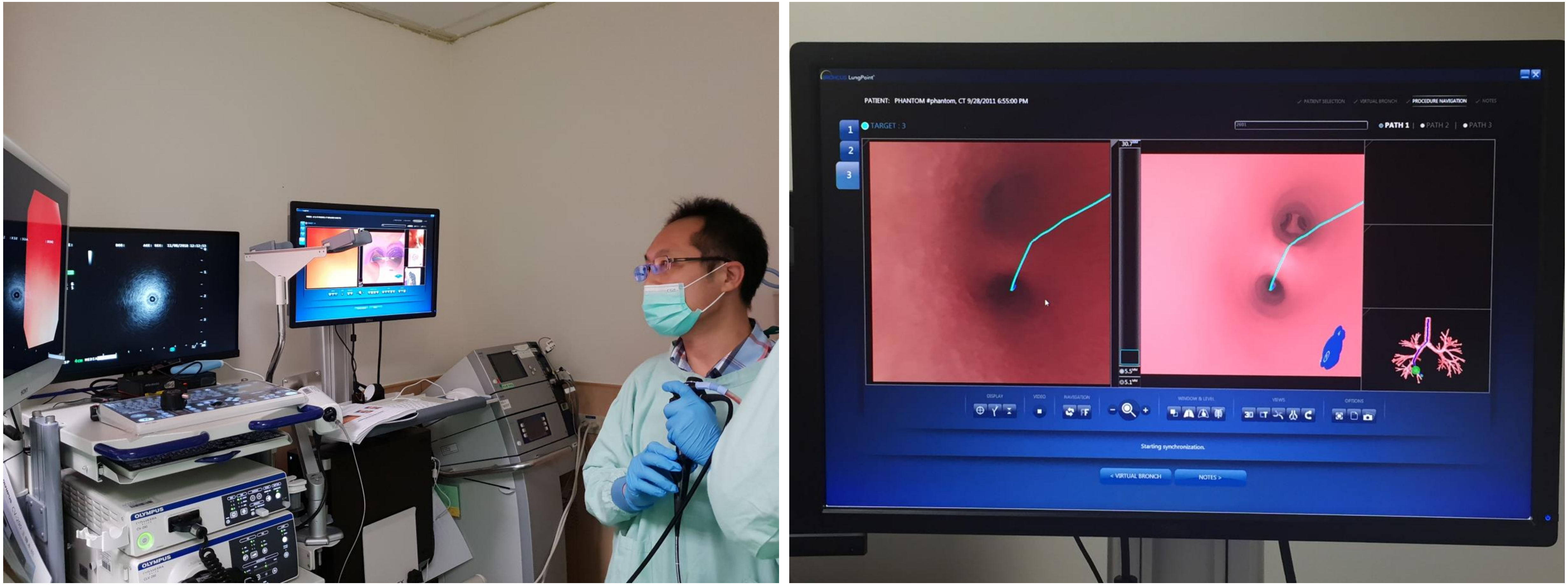

Figure S4. The Electromagnetic navigation image of superDimensionTM System

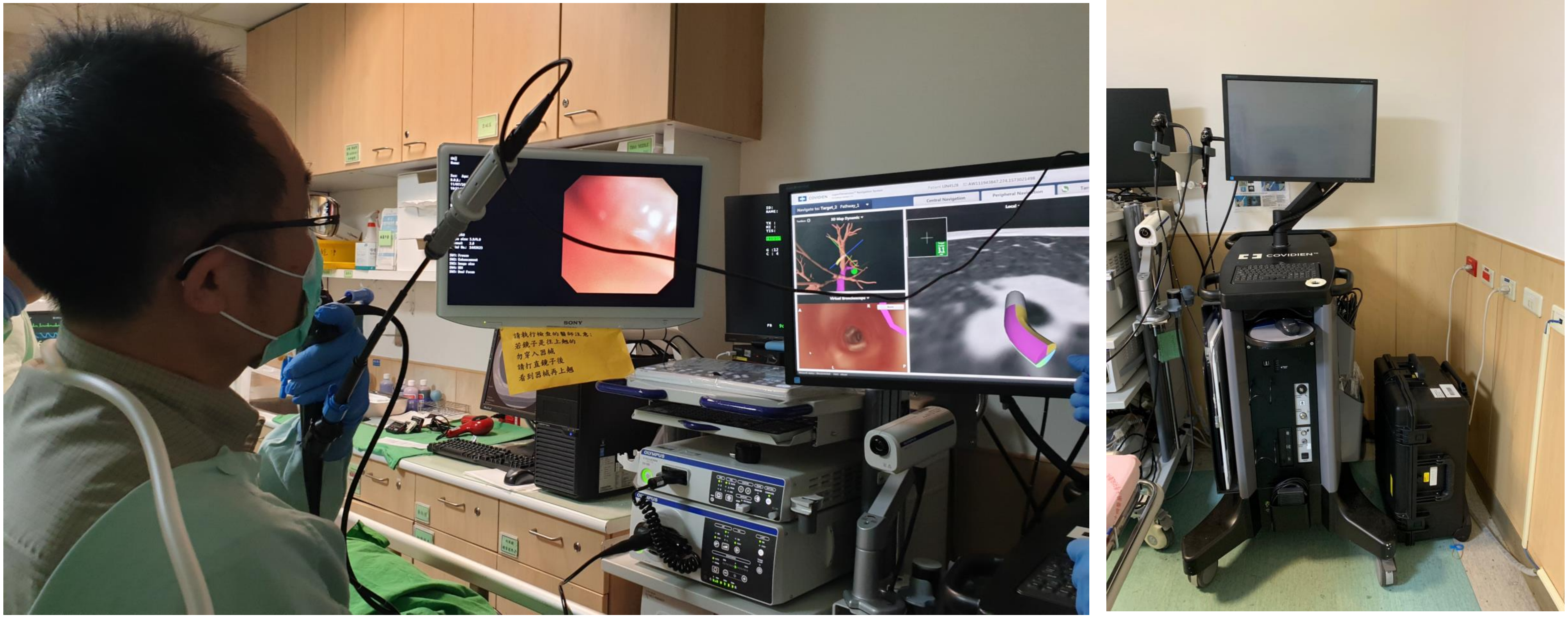

Figure S5. The Cone-beam computed tomography

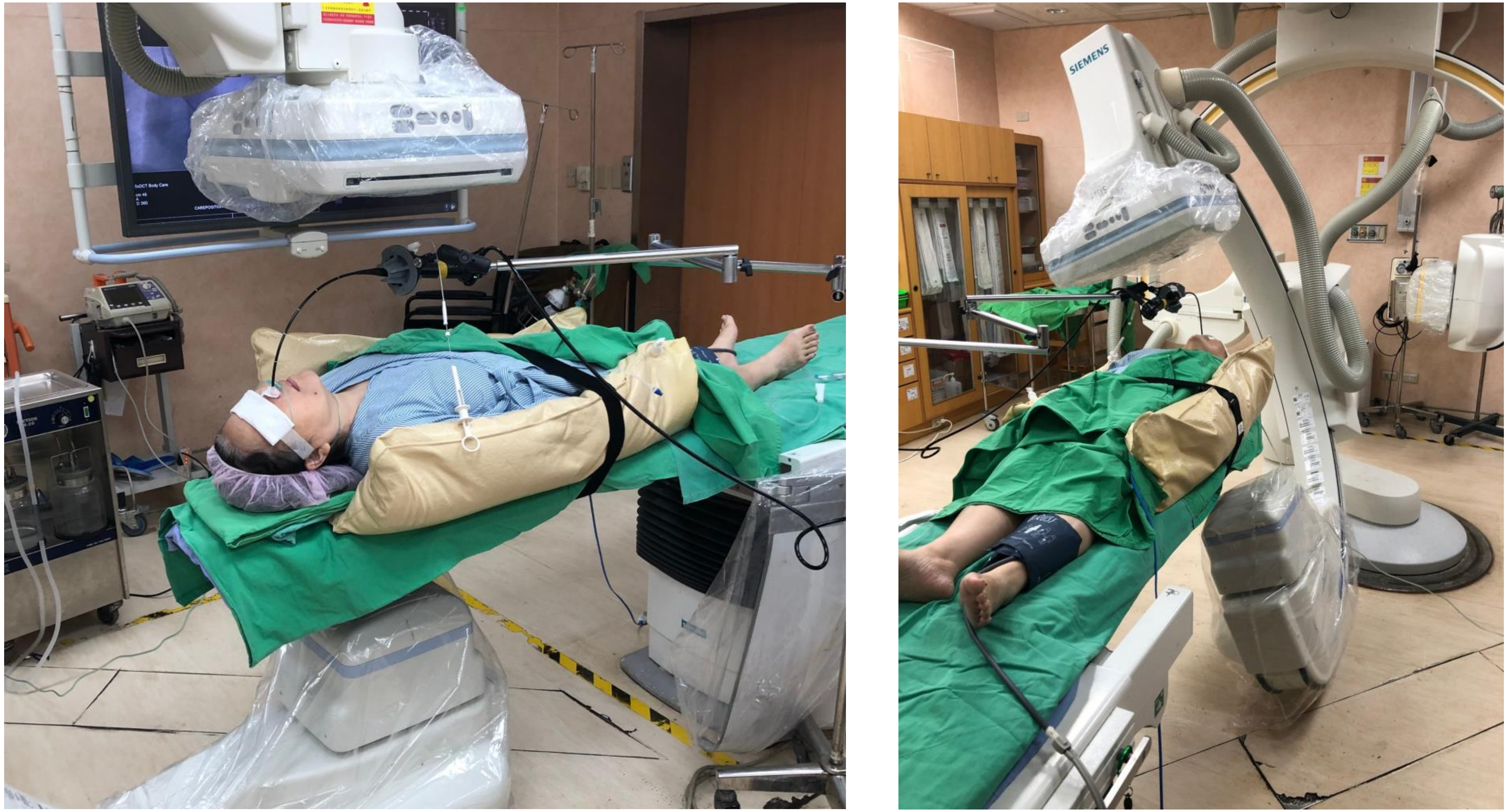

Figure S6. The image of augmented fluoroscopy

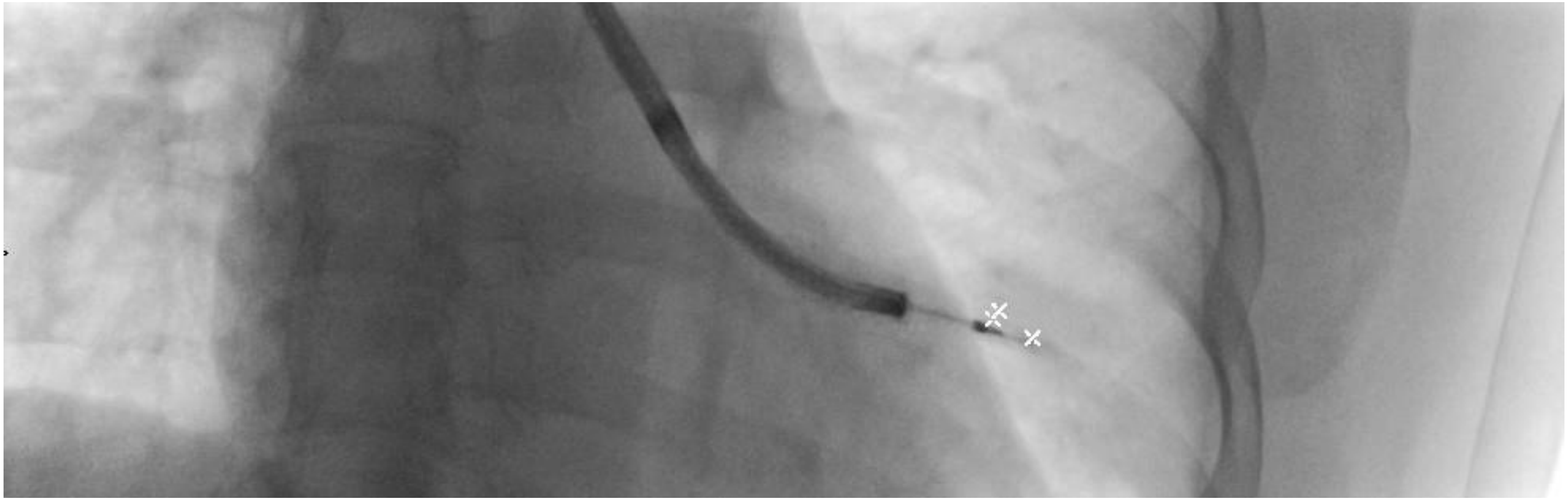

Supplement: Supplementary file 1 [file diagnostics-11-01984-s001.zip › diagnostics-1419757-supplementary.pdf]
